# White Matter Integrity Is Associated With the Amount of Physical Activity in Older Adults With Super-aging

**DOI:** 10.3389/fnagi.2020.549983

**Published:** 2020-09-16

**Authors:** Bori R. Kim, Hunki Kwon, Min Young Chun, Kee Duk Park, Soo Mee Lim, Jee Hyang Jeong, Geon Ha Kim

**Affiliations:** ^1^Department of Neurology, Ewha Womans University Mokdong Hospital, College of Medicine, Ewha Womans University, Seoul, South Korea; ^2^Ewha Medical Research Institute, Ewha Womans University, Seoul, South Korea; ^3^Department of Neurology, Yale University School of Medicine, New Haven, CT, United States; ^4^Department of Radiology, Ewha Womans University Seoul Hospital, College of Medicine, Ewha Womans University, Seoul, South Korea

**Keywords:** diffusion tensor imaging, white matter integrity, cognitive function, physical activity, superAgers

## Abstract

Previous studies have introduced the concept of “SuperAgers,” defined as older adults with youthful memory performance associated with the increased cortical thickness of the anterior cingulate cortex. Given that age-related structural brain changes are observed earlier in the white matter (WM) than in the cortical areas, we investigated whether WM integrity is different between the SuperAgers (SA) and typical agers (TA) and whether it is associated with superior memory performance as well as a healthy lifestyle. A total of 35 SA and 55 TA were recruited for this study. Further, 3.0-T magnetic resonance imaging (MRI), neuropsychological tests, and lifestyle factors related to cognitive function, such as physical activity and duration of sleep, were evaluated in all participants. SA was defined as individuals demonstrating the youthful performance of verbal and visual memory, as measured by the Seoul Verbal Learning Test (SVLT) and the Rey-Osterrieth Complex Figure Test (RCFT), respectively. Tract-based spatial statistics (TBSS) analysis was used to compare the diffusion values such as fractional anisotropy (FA), mean diffusivity (MD), radial diffusivity (RD) and axial diffusivity (AD) between the SA and TA. SA exhibited better performance in memory, attention, visuospatial, and frontal executive functions than the TA did. SA also exhibited greater amounts of physical activity than the TA did. As compared to TA, SA demonstrated higher FA with lower MD, RD, and AD in the corpus callosum and higher FA and lower RD in the right superior longitudinal fasciculus (SLF), which is significantly associated with memory function. Interestingly, FA values of the body of corpus callosum were correlated with the amount of physical activity. Our findings suggest that WM integrity of the corpus callosum is associated with superior memory function and a higher level of physical activities in SA compared to TA.

## Introduction

Age-related cognitive decline is a common feature that occurs with age (Hedden and Gabrieli, 2004). However, previous research findings suggest that such a decline is not inevitable and have introduced the concept of the “SuperAgers” (Pudas et al., 2013; Rogalski et al., 2013; Gefen et al., 2015; Sun et al., 2016). “SuperAgers” refer to older adults whose cognitive abilities are comparable to those in their middle age (Harrison et al., 2012; Gefen et al., 2015) or young adulthood (Harrison et al., 2018; Zhang et al., 2020).

In recent years, several neuroimaging studies have shown that the cortical thickness or volume in the anterior cingulate cortex of the SuperAgers is greater than that the average elderly population and similar to that in younger adults (Harrison et al., 2012; Rogalski et al., 2013; Gefen et al., 2015). Recent studies have demonstrated that SuperAgers are characterized by high cortical thickness in the medial prefrontal, angular gyrus, cingulate gyrus, and anterior insular areas, which are predominantly located within the default mode and salience networks (Sun et al., 2016; Harrison et al., 2018) widely thought to be affected by age-related atrophy in healthy elderly populations (McGinnis et al., 2011; Fjell et al., 2014).

Notably, aging affects the white matter (WM) and gray matter (Guttmann et al., 1998; Chen et al., 2001). Previous studies have shown that the loss of myelin integrity in the WM is considered to be one of the key mechanisms underlying normal age-related cognitive decline (Salat et al., 2005; Madden et al., 2008, 2012). Also, another study reported only a 10% loss in the total number of neurons in the cortex during normal aging, whereas as much as 28% of WM volume was decreased (Pakkenberg and Gundersen, 1997). Furthermore, the disruption in WM integrity was associated with poor performance on tasks evaluating processing speed, executive function, and episodic memory in normal aging (Gunning-Dixon and Raz, 2000; Bennett and Madden, 2014; Fjell et al., 2017). Given that WM integrity declines progressively during normal aging, it could be more related to age-related cognitive decline than the cortical areas (Morrison and Hof, 1997), so it is important to investigate the topography of WM integrity in SuperAgers since preserved areas of the WM could be one of the most resilient areas in age-related cognitive decline (Madden et al., 2009). Although several neuroimaging studies have investigated cortical structures in SuperAgers (Harrison et al., 2012; Sun et al., 2016), to our knowledge, no studies have yet assessed WM integrity in SuperAgers.

Furthermore, it is well known that high level of physical activity is associated with improved cognitive function (Kraft, 2012) and high integrity of the WM (Tian et al., 2015; Oberlin et al., 2016), while reduced sleep is associated with poor cognitive function (Nebes et al., 2009) and reduced integrity of the WM in older adults (Baillet et al., 2017; Gadie et al., 2017; Sexton et al., 2017). Considering the potential relationship between a healthy lifestyle and WM integrity, one would expect these lifestyle factors to be significantly associated with WM integrity in SuperAgers.

The purpose of this study, therefore, was to investigate whether SuperAgers have increased WM integrity related to youthful cognitive function and whether a healthy lifestyle such as a high amount of physical activity or sleep time is associated with WM integrity in SuperAgers.

## Materials and Methods

We recruited a total of 90 community-dwelling healthy elderly subjects with normal cognitive function as assessed by the Gangseo Center for Dementia, one of the public facilities for dementia prevention in Seoul. Elderly individuals with normal cognitive function were defined as those aged 60 or above who scored higher than one SD below the mean of age and education-matched norm, on the tests evaluating memory, attention, language, visuospatial, and frontal executive functions in the Seoul Neuropsychological Screening Battery (Kang et al., 2003). We excluded individuals with any of the following characteristics: (1) suspected or confirmed mild cognitive impairment or dementia; (2) suspected or confirmed major neurological or psychiatric illnesses, including major depressive disorders; (3) contraindications to magnetic resonance imaging (MRI); (4) visual or hearing impairments severe enough to interfere with questionnaire response; (5) history of medications that could affect cognitive and emotional functions in the last 3 months; and or (6) other major medical problems. The definition of SuperAgers was based on memory performance and was determined by performance at or above average normative values of middle-aged adults (45 years old) on the tests of delayed recall in both the Seoul Verbal Learning Test (SVLT) and the Rey-Osterrieth Complex Figure Test (RCFT). Written informed consent was obtained from all participants, and the study was approved by the Institutional Review Board of Ewha Woman’s University Mokdong Hospital (IRB approval number: 2017-12-047).

### Neuropsychological Assessments

All participants were administered a standardized neuropsychological battery called the Seoul Neuropsychological Screening Battery (SNSB), which has been previously described in detail (Kang et al., 2003). Cognitive evaluations included seven tests covering five domains: attention (forward and backward digit span), language functions (short form of the Korean version of the Boston Naming Test), visuospatial functions (scores of copying test from the RCFT), memory functions (delayed recall of SVLT and RCFT), and frontal executive functions (Digit Symbol Coding). All neuropsychological data were analyzed using the standardized *z* score of each domain.

### MR Image Acquisition

All participants underwent brain MRI using the same imaging protocols and the same MRI scanner (Achieva, Philips 3.0T; Eindhoven, Netherlands). The 3D T1 turbo field echo MR images were acquired with the following imaging parameters: sagittal slice thickness, 0.5 mm; no gap; repetition time (TR), 9.9 ms; echo time (TE), 4.6 ms; flip angle, 8°; and a matrix size of 240 × 240 pixels. Fluid attenuated inversion recovery (FLAIR) MR images were used to evaluate abnormal white matter hyperintensities (WMH). These were assessed using the following imaging parameters: axial slice thickness, 2 mm; no gap; TR, 11,000 ms; TE, 125 ms; inversion time (TI), 2,800 ms; matrix size, 256 × 189 pixels. For tract-based spatial statistics (TBSS), diffusion tensor images (DTI) were acquired using a diffusion-weighted single-shot echo-planar imaging sequence with the following parameters: TR, shortest ms; TE, 91 ms; flip angle, 90°; b-factor, 1,000 s/mm^2^; matrix size, 128 × 126; 70 axial sections; slice thickness, 2 mm; in-plane resolution, 1.72 × 1.72 mm. With the baseline image without diffusion weighting (the reference volume), diffusion-weighted images were acquired from 32 different directions.

### Tract-Based Spatial Statistics

DTI data were processed using the FMRIB Software Library (FSL 6.0.2. release)^1^, FMRIB’s Diffusion Toolbox and TBSS analysis (TBSS) was (Smith et al., 2004, 2006). To improve the image quality, eddy current-induced distortion and motion artifacts were corrected using the affine alignment of each diffusion-weighted image to the non-diffusion weighted volume (b0 image) using the FMRIB Linear Image Registration Tool (Smith, 2002; Jenkinson et al., 2012). The b vectors were then rotated appropriately using the “fdt-rotate-bvecs” tool as part of FSL (Smith et al., 2004; Leemans and Jones, 2009). Diffusion tensors were then calculated at the level of an individual voxel to generate fractional anisotropy (FA) images using DTIfit, and the images of brain tissue were subsequently extracted using the Brain Extraction Tool (Smith, 2002) from the FSL. All subjects’ FA data were realigned onto the standard FMRIB58_FA template provided by the FSL software using a nonlinear registration algorithm implemented in the TBSS package. The registered FA images were averaged to generate a skeletonized mean FA image, which represents the main fiber tracts and the center of all tracts common to the group. A threshold of 0.3 was applied to exclude peripheral tracts where there was significant inter-subject variability and/or partial volume effect due to gray matter. Each participant’s aligned FA data were projected onto the mean skeleton to create a skeletonized FA map. A similar process was performed for the mean diffusivity (MD), axial diffusivity (AD), and radial diffusivity (RD). Each participant’s movement in *x*, *y*, and *z* coordinates was based on the output of eddy_correct. The intracranial volume (ICV) of each participant was calculated using an automated process with the MRI_segstats command in FreeSurfer^2^ (for more details).

### Physical Activity and Total Sleep Time

Since the amount of physical activity and sleep is associated with cognitive function and WM integrity in the elderly (Wassenaar et al., 2019), all participants wore a Fitbit Alta HR (Fitbit Inc., San Francisco, CA, USA) for a week to measure both physical activity and total sleep time (TST). Recent studies using the Fitbit have demonstrated that the Fitbit accurately measures physical activity and TST (Stahl and Insana, 2014; Feehan et al., 2018; Tedesco et al., 2019). We used the physical activity energy expenditure (PAEE) measure, which includes low-intensity physical activity (e.g., housework and daily chores) as well as moderate and vigorous physical activity (e.g., jogging and walking; Donahoo et al., 2004; Middleton et al., 2011), and TST that decrease with aging (Campbell and Murphy, 2007). The PAEE and TST were obtained from the Fitbit device itself (Chowdhury et al., 2017; Lee et al., 2019) and were calculated using the Fitbit website^3^ (Murakami et al., 2019). Of the 90 participants, Fitbit data from 11 participants were missing due to technical errors in uploading data from the device to the Fitbit website: PAEE (one SuperAger and four Typical Agers) and TST (two SuperAgers and four Typical Agers).

### Visual Rating of White Matter Hyperintensities

White matter hyperintensity (WMH) on FLAIR images was assessed by one neurologist using the modified Fazekas scale, which was validated in a previous study (Noh et al., 2014). On this scale, periventricular WMH was classified as P1 (cap and band <5 mm), P2 (5 mm ≤ cap or band <10 mm), and P3 (10 mm ≤ cap or band), whereas deep WMH was classified as D1 (maximum diameter of deep WM lesion <10 mm), D2 (10 mm ≤ lesion < 25 mm), and D3 (≥25 mm).

Hyperintensities evident in the axial slice just above the lateral ventricles were considered to be periventricular WM lesions, while hyperintensities evident in the second or axial slices above the lateral ventricles were considered to be deep WM lesions. The reliability of this WMH visual rating scale was shown to be quite high (intraclass correlation coefficient between 0.726 and 0.905; Moon et al., 2011). The results were combined to give a final WMH classification of minimal, moderate, or severe, which was encoded as 1, 2, and 3, respectively. Combinations of D1 with P1 (D1P1) and D1 with P2 (D1P2) were classified as “minimal.” D2P1, D3P1, D2P2, D3P2, D1P3, and D2P3 combinations were classified as “moderate,” whereas D3P3 was classified as “severe.”

### Statistical Analyses

Statistical analyses were performed using SPSS 22 (IBM Corp, Armonk, NY, USA). Group differences in demographic data were compared using the chi-square test for categorical variables, independent-sample *t*-tests for continuous variables, and the Mann–Whitney *U* test for non-normal variables. The general linear model was used to assess the group differences in the neuropsychological measures and lifestyle factors with age, sex, and years of education as covariates.

The diffusion maps (i.e., FA, MD, RD, and AD maps) from the TBSS analyses were used for the voxel-wise analysis of group differences between the SuperAgers and Typical Agers. Voxel-wise statistics were conducted with 5,000 permutations using the FSL randomize program^4^ (Nichols and Holmes, 2002). To reduce the effects of excessive head motions during DTI acquisition on the results, translational and rotational movements of each participant were also included as covariates. The general linear model was used to examine the group differences in global and regional diffusion values, adjusting for age, sex, years of education, ICV, Body mass index (BMI), head motions, and WMH as covariates. The results of each group were corrected for multiple comparisons using Monte Carlo simulation with 10,000 iterations implemented in the AFNI’s AlphaSim software.^5^ The threshold of a combination of a voxel-wise *P* < 0.01 with a cluster size of at least 760 mm^3^ was used to correct for multiple comparisons at *P* < 0.05. To identify the location of WM tracts displaying the differences between the two groups, we used the ICBM-DTI-81 WM labels atlas and the JHU tractography atlas in FSL (Hua et al., 2008; Jenkinson et al., 2012). Then, the diffusion values from the significant clusters between the two groups were extracted for further *post hoc* analyses (Table 2). Partial correlation was used to assess the association between the extracted values of diffusion maps and each cognitive domain score or between the extracted values and lifestyle factors (PAEE and TST), adjusting for the same covariates as above. Statistical significance was defined as two-tailed* P* < 0.05; Bonferroni correction was used for multiple testing of five cognitive domains.

**Table 1:** Demographic and clinical characteristics of participants.

	SuperAger (*N* = 35) Mean (SD)	Typical Ager (*N* = 55) Mean (SD)	*P*-value
Demographic characteristics
Age (years)	71.0 (5.3)	73.0 (5.6)	0.10
Education (years)	9.8 (3.8)	10.9 (3.4)	0.14
Female, *n* (%)	29.0 (82.9)	46.0 (83.6)	0.92
BMI (kg/m^2^)	24.5 (3.2)	24.5 (3.3)	0.99
Diabetes mellitus, *n* (%)	4.0 (11.4)	13.0 (23.6)	0.15
Hypertension, *n* (%)	12.0 (34.3)	29.0 (52.7)	0.09
Hyperlipidemia, *n* (%)	9.0 (25.7)	14.0 (25.5)	0.98
Cognitive performance (*z*-scores)^a^
Attention	0.1 (1.8)	-0.9 (1.2)	<0.001**
Visuospatial	0.2 (0.6)	-0.1 (0.8)	0.02
Memory	2.2 (1.3)	-0.2 (1.1)	<0.001**
Language	0.3 (0.8)	0.1 (0.9)	0.19
Frontal executive	1.5 (1.3)	0.6 (1.0)	0.001**
Lifestyle factors ^b^
*Physical activity*			
Energy expenditure (kcal/min)	903.8 (368.7)	725.1 (180.6)	0.01*
*Sleep*			
Total sleep time (min)	357.0 (47.5)	350.4 (51.7)	0.63
Neuroimaging characteristics^b^
Intracranial volume (cm^3^)	156.4 (14.8)	147.6 (13.9)	0.001*
*White matter hyperintensities*			
Fazekas	1.2 (0.6)	1.3 (0.6)	0.78
*Head movements*
Translation (mm)	1.7 (0.2)	1.8 (0.2)	0.13
Rotation (degree)	0.3 (0.2)	0.3 (0.2)	0.86

Data are presented as mean (SD, standard deviation) or number (%) for dichotomous variables. BMI, body mass index. WM, white matter. ^a^*P*-values adjusted for age, gender, and education and corrected for multiple testing for five domains of cognitive performance, **P < 0.01 as significant. ^b^Adjusted for age, gender, and education, *P < 0.05 as significant.

**Table 2:** Significant clusters of differences between the SuperAgers (SA) and Typical Agers (TA).

				MNI atlas coordinates			
		White-matter tracts JHU white-matter tractography atlas	Number of voxels in the cluster	(location of maximum *t*-value)			
				*x*	*y*	*z*	*P*
FA (SA > TA)	-	Genu of the corpus callosum	273	14	27	17	<0.001
	-	Body of corpus callosum	234	1	−6	26	<0.001
	R	Superior longitudinal fasciculus	117	52	3	23	<0.001
	-	Forceps minor	97	−20	40	22	0.007
MD (SA < TA)	-	Body of corpus callosum	370	6	10	22	<0.001
	L	Superior longitudinal fasciculus	218	−20	−15	41	<0.001
	-	Splenium of the corpus callosum	136	−8	−35	22	<0.001
	L	Inferior longitudinal fasciculus	120	−34	−32	4	0.002
RD (SA < TA)	-	Body of corpus callosum	328	6	11	23	<0.001
	-	Genu of the corpus callosum	274	14	27	18	<0.001
	L	Inferior longitudinal fasciculus	188	−34	−32	4	<0.001
	L	Superior longitudinal fasciculus	129	−20	−15	40	0.002
	R	Superior longitudinal fasciculus	106	18	34	34	<0.001
AD (SA < TA)	-	Genu of the corpus callosum	136	3	22	16	<0.001
	-	Splenium of the corpus callosum	102	−8	−35	22	<0.001

*Significant clusters were calculated by the tract-based spatial statistics (TBSS) analysis for the group-differences in FA, MD, RD, and AD values after adjustment for age, sex, education, intracranial volume, body mass index, translation, rotation, and WM hyperintensities. Abbreviations: R, right; L, left; FA, fractional anisotropy; MD, mean diffusivity; RD, radial diffusivity; AD, axial diffusivity; MNI, Montreal Neurological Institute*.

## Results

### Participant Characteristics

The demographic and clinical characteristics of all participants are summarized in Table 1. The SuperAgers and Typical Agers showed no statistically significant difference in terms of age (*t* = −1.7, *P* = 0.10), sex (*χ^2^* = 0.01, *P* = 0.92), year of education (Mann–Whitney *U* = 788.0, *P* = 0.14), and BMI (*t* = 0.01, *P* = 0.99). SuperAgers demonstrated greater PAEE (*z* = 2.5, *P* = 0.01) relative to Typical Agers, whereas no significant difference in TST was observed between the two groups (*z* = 0.5, *P* = 0.63).

### Neuropsychological Performance

SuperAgers showed better performance not only in memory (*z* = 10.1, *P* < 0.001) but also in attention (*z* = 3.5, *P* < 0.001) and frontal executive functions (*z* = 3.5, *P* = 0.001) than Typical Agers (Table 1). However, there was no significant difference in terms of language (*z* = 1.3, *P* = 0.19) and visuospatial function (*z* = 2.4, *P* = 0.02).

### Differences in WM Integrity Between the SuperAgers and Typical Agers

**Figure 1:**
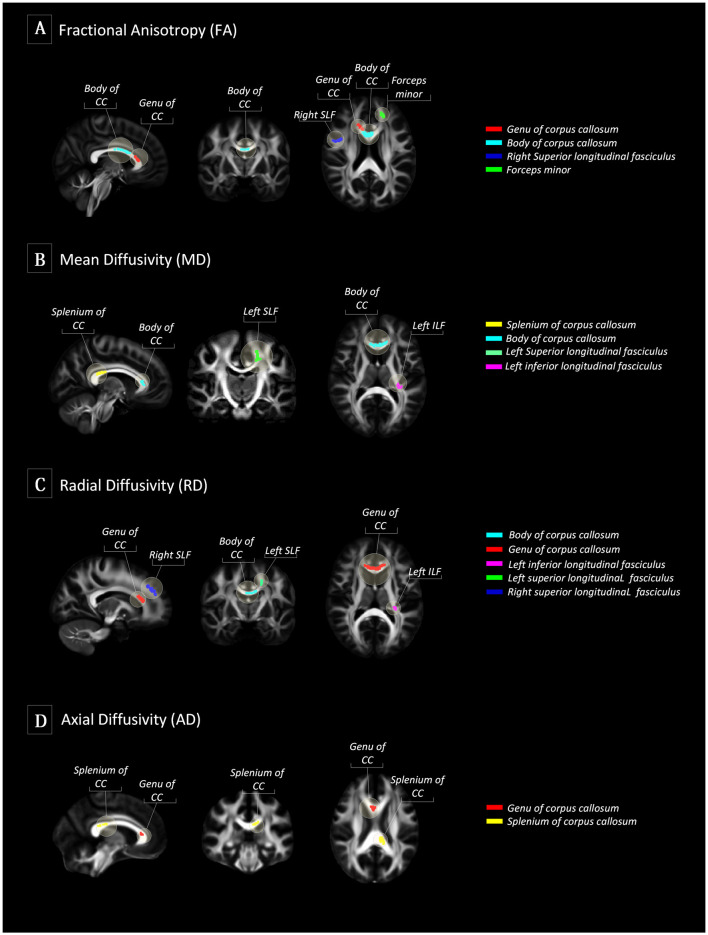
Tract-based spatial statistics (TBSS) between SuperAgers and Typical Agers. **(A)** In terms of fractional anisotropy (FA) values, SuperAgers had significantly higher FA in the genu of the corpus callosum, the body of corpus callosum, right superior longitudinal fasciculus (SLF), and forceps minor compared to Typical Agers. **(B)** SuperAgers showed significantly lower mean diffusivity (MD) in the body and splenium of the corpus callosum as well as left SLF and inferior longitudinal fasciculus (ILF) compared to Typical Agers. **(C)** SuperAgers showed lower radial diffusivity (RD) values in the genu/body of corpus callosum as well as bilateral SLF and the left ILF compared to Typical Agers. **(D)** Lower axial diffusivity (AD) values in the genu and the splenium of the corpus callosum were shown in the SuperAgers relative to Typical agers. There were no significant areas that showed higher FA or lower mean diffusivity (MD), axial diffusivity (AD), and radial diffusivity (RD) in Typical Agers relative to SuperAgers.

There were no differences in global FA (0.51 ± 0.02 for Typical Agers vs. 0.52 ± 0.02 for SuperAgers, *P* = 0.37), MD (0.0007 ± 0.00002 vs. 0.0007 ± 0.00002, *P* = 0.08), RD (0.0005 ± 0.00002 vs. 0.0005 ± 0.00001, *P* = 0.15), and AD (0.001 ± 0.00002 vs. 0.001 ± 0.00003, *P* = 0.10) values between the SuperAgers and Typical Agers. However, SuperAgers had significantly higher FA in the genu of the corpus callosum, the body of corpus callosum, right superior longitudinal fasciculus (SLF), and the forceps minor than the Typical Agers (cluster-corrected *P* < 0.05). Also, the SuperAgers showed decreased MD in the body and splenium of the corpus callosum as well as the left SLF and inferior longitudinal fasciculus (ILF; cluster-corrected *P* < 0.05; Table 2, Figure 1). SuperAgers also showed lower RD values in the corpus callosum, left ILF, and bilateral SLF and lower AD values in the corpus callosum than the Typical Agers (Table 2, Figure 1). There were no significant areas that showed higher FA or lower MD, RD, and AD in Typical Agers than in SuperAgers.

### Relationship Between the Diffusion Values and Cognitive Performance

**Figure 2:**
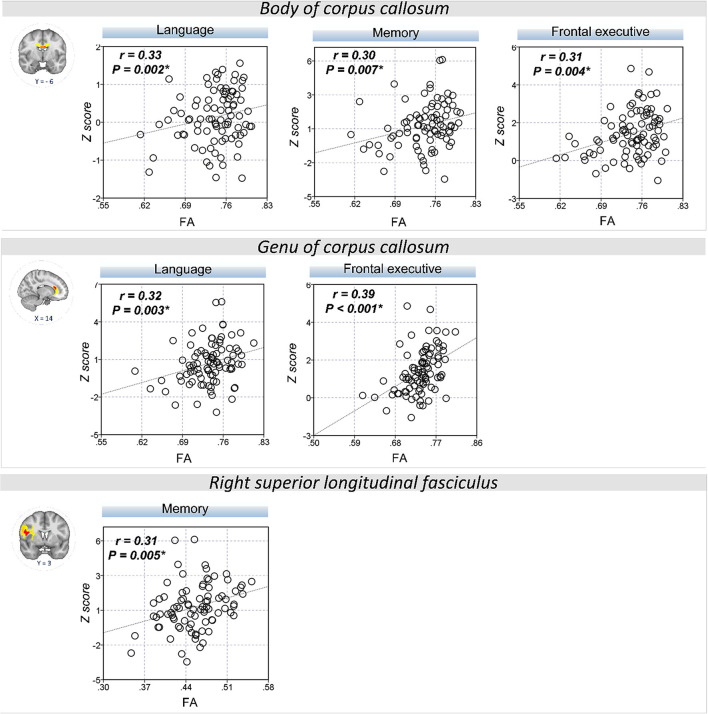
Correlation between FA values and cognitive performance. A scatter plot indicates a significant positive correlation between the FA values of the body of corpus callosum and memory, language, and frontal executive function. Also, the FA values of the genu of the corpus callosum were correlated with language and the frontal executive function while those of the right SLF was correlated with the memory functions. *P < 0.01 as significant.

**Figure 3:**
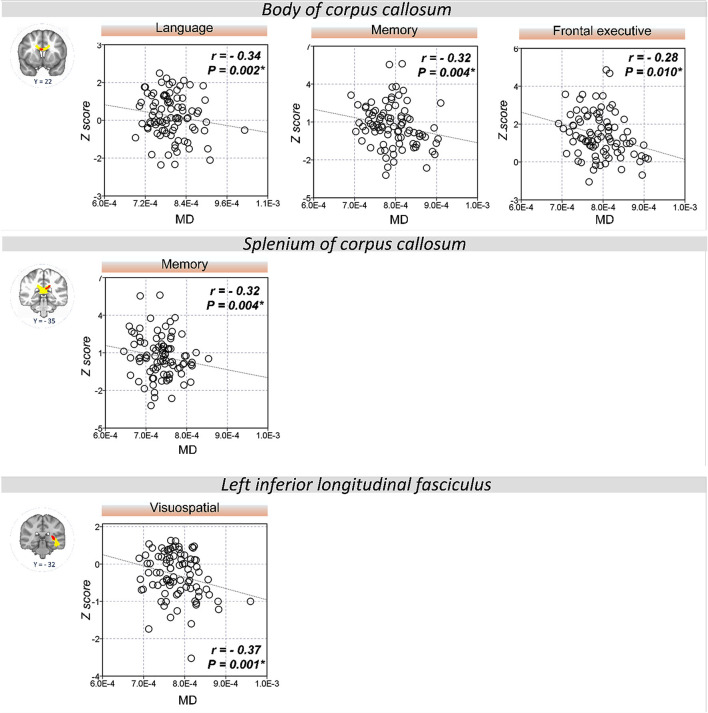
Correlation between MD values and cognitive performance. A significant correlation was shown between the MD values of the body of corpus callosum and memory, language, and frontal executive function. Also, the MD values of the splenium of the corpus callosum and left ILF were correlated with memory and visuospatial functions, respectively. *P < 0.01 as significant.

The extracted FA and MD values from the body of corpus callosum were correlated with memory (*r* = 0.30, *P* = 0.007 for FA; *r* = −0.32, *P* = 0.004 for MD), language (*r* = 0.33, *P* = 0.002 for FA; *r* = −0.34, *P* = 0.002 for MD), and frontal executive function (*r* = 0.31, *P* = 0.004 for FA; *r* = −0.28, *P* = 0.01 for MD; Figures 2, 3). The FA values of genu of corpus callosum were correlated with language (*r* = 0.32, *P* = 0.003) and frontal executive function (*r* = 0.39, *P* < 0.001), while those of the right SLF were correlated with memory functions (*r* = 0.31, *P* = 0.005; Figure 2). In addition, the MD values of the splenium of corpus callosum and left ILF were correlated with memory (*r* = −0.32, *P* = 0.004) and visuospatial functions (*r* = −0.37, *P* = 0.001; Figure 3). The significant correlations between RD or AD values with cognitive function are shown in Supplementary Figures S1, S2, which also indicated that the RD values of the body of corpus callosum (*r* = −0.31, *P* = 0.005) and right SLF (*r* = −0.31, *P* = 0.005) were significantly associated with memory. Furthermore, the AD values of the genu of corpus callosum was correlated with memory (*r* = −0.29, *P* = 0.008) while those of the splenium of corpus callosum were associated with memory (*r* = −0.32, *P* = 0.004) and attention (*r* = −0.30, *P* = 0.008).

### Correlation Between the Diffusion Values and Physical Activity or Total Sleep Time

**Figure 4:**
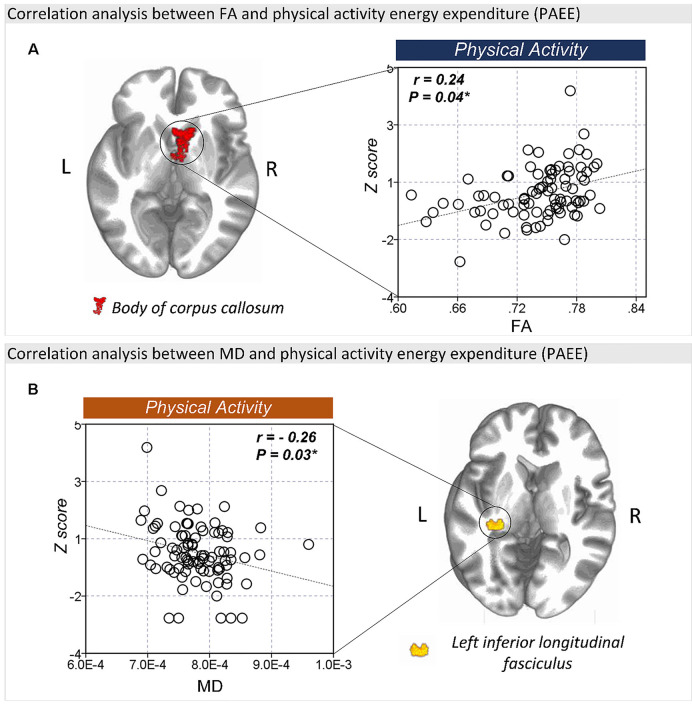
The correlation between FA and lifestyle factors. **(A)** There was a significant correlation between the FA values of the body of corpus callosum and physical activity energy expenditure (PAEE). **(B)** The MD values of the left ILF were also significantly correlated with PAEE. *P < 0.05 as significant.

The FA values in the body of corpus callosum (*r* = 0.24, *P* = 0.04) was positively correlated with PAEE (Figure 4A), while the MD (*r* = −0.26, *P* = 0.03; Figure 4B) and RD (*r* = −0.32, *P* = 0.01, Supplementary Figure S3) values of the left ILF were negatively correlated with PAEE. However, there were no significant correlation between the PAEE and the AD values of the genu of corpus callosum (*r* = −0.09, *P* = 0.43) or the splenium of corpus callosum (*r* = −0.14, *P* = 0.22). Furthermore, there were no significant correlations between the diffusion values and TST either.

## Discussion

In this study, we found that SuperAgers had greater WM integrity in the corpus callosum as well as the SLF and ILF than the Typical Agers, which was associated with enhanced cognitive function as well as with the high amount of physical activity.

In the current study, the SuperAgers showed better performance in memory, attention, and frontal executive functions than the Typical Agers. This finding was in line with the previous findings showing that SuperAgers showed higher performance in memory, working memory, and processing speed than Typical Agers. This finding is also consistent with the previous findings of a longitudinal study reporting that SuperAgers maintained stable cognitive performance not only in memory but also in non-memory domains (Gefen et al., 2014). There are two explanations for the increased cognitive functions in SuperAgers: (1) the high cognitive performance in the SuperAgers may reflect a high baseline cognitive function since their youth; or (2) the cognitive function in SuperAgers is related to resilience against age-related decline. Considering the former hypothesis, the SuperAgers would have had excellent cognitive capabilities in their earlier lives. However, given that no difference in premorbid IQ between the SuperAgers and Typical Agers was reported in previous studies (Sun et al., 2016; Cook et al., 2017), we suggest that the SuperAgers are resistant to age-related cognitive changes. Since it is impossible to know if the SuperAgers in the current study had been superior performers in their youth, a longitudinal examination is needed to investigate whether the SuperAgers are resistant to the age-related cognitive decline.

One of the main findings of this study was that the SuperAgers had higher FA and lower MD, RD, and AD values in the corpus callosum than the Typical Agers. Also, SuperAgers demonstrated higher FA and lower RD in the genu and body of corpus callosum as well as the right SLF compared to the Typical Agers. Previous studies demonstrated that age-related reduction in FA occurred in conjunction with the increase in MD, RD, and AD, specifically in the body of corpus callosum and SLF (Burzynska et al., 2010; Madden et al., 2012). The lower FA values with high RD and/or high MD and AD may suggest age-related WM degeneration or demyelination in older adults (Burzynska et al., 2010; Madden et al., 2012), so our findings suggest that SuperAgers had less age-related WM degeneration or demyelination in those areas than Typical Agers. In line with previous findings, the current finding of high WM integrity observed in SuperAgers in areas such as the corpus callosum and SLF, indicating that age-related degeneration is particularly prevalent in anterior to posterior (Sullivan and Pfefferbaum, 2006) and superior to inferior (Sexton et al., 2014) gradients of greater to lower vulnerability, can be backed by the retrogenesis hypothesis stating that late-myelinating tracts are the first to degenerate with aging (Bartzokis, 2004; Brickman et al., 2012).

It should be noted that the FA and the MD values in the body of corpus callosum were correlated with memory, language, and frontal executive function. In addition, the FA and the RD values of the right SLF were also correlated with memory function. Given that the integrity of WM tracts has been associated with various cognitive functions (Madden et al., 2012) and loss of microstructural integrity of WM has been thought to contribute to age-related cognitive decline (Voineskos et al., 2012), it could be plausible that greater integrity of WM tracts play an important role in the performance of cognitive functions, including memory and frontal executive functions in SuperAgers.

The concept of SuperAgers overlaps with the concept of cognitive reserve. Therefore, it could be possible that the high cognitive performance in SuperAgers is reflective of high cognitive reserve. The concept of cognitive reserve has recently been suggested to explain individual differences in trajectories of cognitive decline (Stern, 2009). Cognitive reserve is defined as the ability to optimize or maximize performance through differential recruitment of brain networks, which may reflect the ability to mitigate the effects of cognitive decline by aging or age-related diseases (Cabeza et al., 2018). Therefore, individuals with a high cognitive reserve may display greater cognitive performance despite equivalent age-related changes (i.e., presence of atrophy) than individuals with low cognitive reserve (Stern, 2012). However, the SuperAgers construct describes a phenotype of preserved cognitive function in older age that may reflect unique neurobiological characteristics (i.e., increased cortical thickness or volume; Dang et al., 2019). Therefore, the high cognitive reserve hypothesis does not appear to be relevant to the SuperAgers since high cognitive performance in SuperAgers was associated with the integrity of WM in the current study. Also, an important proxy measure for the cognitive reserve is the educational level (Stern, 2003). Given that no difference in years of education was observed between the SuperAgers and Typical Agers, the level of the cognitive reserve might not be different between the two groups. However, since the cognitive reserve is a latent construct that cannot be directly measured (Opdebeeck et al., 2016), different methods of estimating cognitive reserve such as occupational attainment or cognitive-stimulating activities could lead to findings different from ours.

The SuperAgers showed higher amounts of physical activity than Typical Agers. This observation is compatible with that of previous studies showing that older adults classified as “resilient-agers,” who had comparable processing speed as younger adults, reported a higher level of physical activity than older adults of the same age (Bott et al., 2017). Also, we found that the FA values of the body of corpus callosum as well as the MD values of the left ILF were significantly correlated with the amount of physical activity. This finding is also in line with previous results, which showed that a high level of physical activity is associated with reduced age-related loss of WM integrity in older adults (Gow et al., 2012; Johnson et al., 2012; Oberlin et al., 2016). Given that the amount of physical activity had a protective effect against the age-related damage of the WM tracts such as the corpus callosum (Strömmer et al., 2018), our findings suggest that the WM integrity in the SuperAgers was related to high levels of physical activity.

Unexpectedly, there were no differences in the TST between the SuperAgers and Typical Agers. Also, there was no significant correlation between WM integrity and TST either. Since there is a myriad of evidence that sleep deprivation or inadequate sleep contributes to cognitive deficits (Nebes et al., 2009; Yaffe et al., 2014), we expected the SuperAgers to have longer sleep duration as well as greater WM integrity associated with TST than the Typical Agers. This lack of difference in TST between the groups could be explained by the optimal dose model of sleep (Marshall et al., 2008), which proposed that a specified amount of sleep is necessary for optimal health and functioning and that sleeping above or below the specified limit is detrimental. Given that both SuperAgers and Typical Agers are cognitively unimpaired older adults, it could be possible that both groups had maintained adequate time of sleep, which resulted in the lack of difference in TST between them. A previous study showed that there was no dose-relationship between sleep duration and cognitive performance in older adults (Richards et al., 2017).

There were several limitations to the current study. First, this was a cross-sectional study; therefore, it was difficult to derive a causal relationship between the diffusion values and high cognitive function in SuperAgers. Second, although we found that the diffusion values of the corpus callosum and several association fibers were positively correlated with the amount of physical activity, it was not a causal relationship either. Furthermore, 1 week of physical activity or sleep could not represent an individual’s lifelong lifestyle. Given that SuperAgers can be the product of a lifelong process involving complex and multiple factors throughout the lifetime, it is important to measure a lifelong lifestyle. Since we had limited information on the lifestyle of the participants in their youth because of the cross-sectional design, it should be noted that the significant correlation between the WM integrity and amount physical activity can only indicate that the WM integrity in SuperAgers is related to the amount of physical activity in their later life rather than to lifelong physical activity. Therefore, a longitudinal study related to a lifelong lifestyle and its effects age-related changes in SuperAgers is warranted to assess this association further. Fourth, although it is well known that genetic susceptibility, such as APOE, could affect WM integrity in SuperAgers (Rogalski et al., 2013), genetic factors were not evaluated in this study. Fifth, one of the important lifestyles related to cognitive function is lifelong engagement in mentally-stimulating activities, such as occupational attainment and leisure activities (Stern and Munn, 2010), which was not investigated in this study either. Sixth, although the original concept of SuperAgers refers to older adults aged 80 years and older who have comparable memory performance of middle-aged individuals, the current study lowered the minimum age criterion to 60 years. Given that the preservation of superior cognition in SuperAgers is relevant to resilience, resistance, and compensation with age, the concept of SuperAgers becomes more meaningful in advanced old age (Rogalski, 2019). Although several recent previous studies related to SuperAgers have also used similar neuropsychological criteria and lower the minimum age criterion of 60 years old (Sun et al., 2016; Bott et al., 2017; Dang et al., 2019), the lowered age-criterion in this study could limit the generalizability of the results in SuperAgers aged 80 years older. Therefore, future studies on SuperAgers in advanced age could be helpful to determine why they can maintain increased cognition and WM integrity. Seventh, the proportion of SuperAgers in this study (37%) was higher than expected. Although the available information regarding the prevalence of SuperAgers in the general population is limited, several previous studies have reported prevalence rates from 17.3% to 42.5% (Sun et al., 2016; Harrison et al., 2018). The high proportion of SuperAgers in this study could be explained by the fact that the study used convenient sampling, recruiting those who are usually healthier and more educated than the general population (Brodaty et al., 2014). Finally, TBSS may have fundamental limitations in analyzing small fiber tracts and regions of crossing fibers or tract junctions (Alexander et al., 2001; Smith et al., 2006; Oouchi et al., 2007; Jones et al., 2013). Therefore, alterations in FA, MD, RD, and AD from TBSS analyses could not always be related to changes in microstructural integrity, which may lead to difficulty in interpreting the results. However, TBSS can still provide useful information on WM and its relationship with behavioral variables.

Nonetheless, this study is the first to show that SuperAgers have greater WM integrity of the corpus callosum and association fibers than Typical Agers and that this observation is associated with increased cognitive functions and physical activity.

## Data Availability Statement

The data used for analyses are available from the corresponding author on reasonable request.

## Ethics Statement

The studies involving human participants were reviewed and approved by the Institutional Review Board of Ewha Womans University Mokdong Hospital (IRB approval number: 2017-12-047). The patients/participants provided their written informed consent to participate in this study.

## Author Contributions

BK was responsible for the conception and design, data analysis, interpretation of the results, and for writing the manuscript. HK was involved in the data analysis and interpretation of the results in the revised manuscript. MC was involved in the collection and assembly of data. KP, JJ, and SL conducted data analysis and interpretation. GK was responsible for the conception and design of the study, administrative support, writing of the manuscript, and final approval of the manuscript.

## Conflict of Interest

The authors declare that the research was conducted in the absence of any commercial or financial relationships that could be construed as a potential conflict of interest.

## Footnotes

^1^http://www.fmrib.ox.ac.uk/fsl

^2^http://surfer.nmr.mgh.harvard.edu/fswiki/eTIV

^3^http://www.fitbit.com

^4^http://fsl.fmrib.ox.ac.uk/fsl/fslwiki/Randomise

^5^http://afni.nimh.nih.gov/afni
