# Supplementary material for: White Matter Integrity Is Associated With the Amount of Physical Activity in Older Adults With Super-aging
Source: Front Aging Neurosci. 2020 Sep 16;12:549983. doi: 10.3389/fnagi.2020.549983 (PMC7525045; doi:10.3389/fnagi.2020.549983)
Supplement: Supplementary file 1 [file Data_Sheet_1.pdf]

Supplementary Material

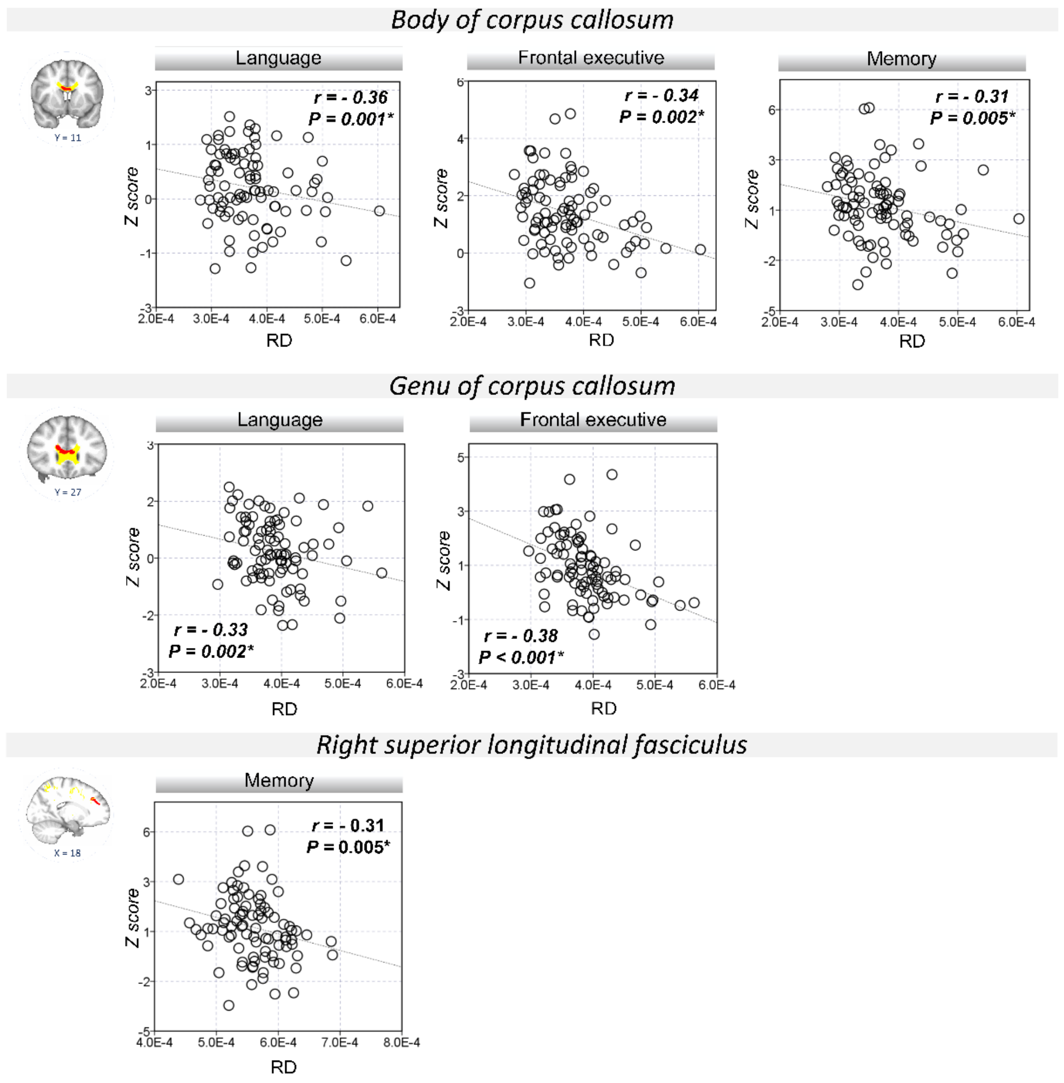

Supplementary Figure S1 | Correlations between radial diffusivity (RD) and cognitive function. There were significant correlations between the RD values in specific white matter tracts and memory, language and frontal executive function after adjusting for age, sex, education, intracranial volume, body mass index, head movements and white matter hyperintensities. *$P < 0.01$ as significant.

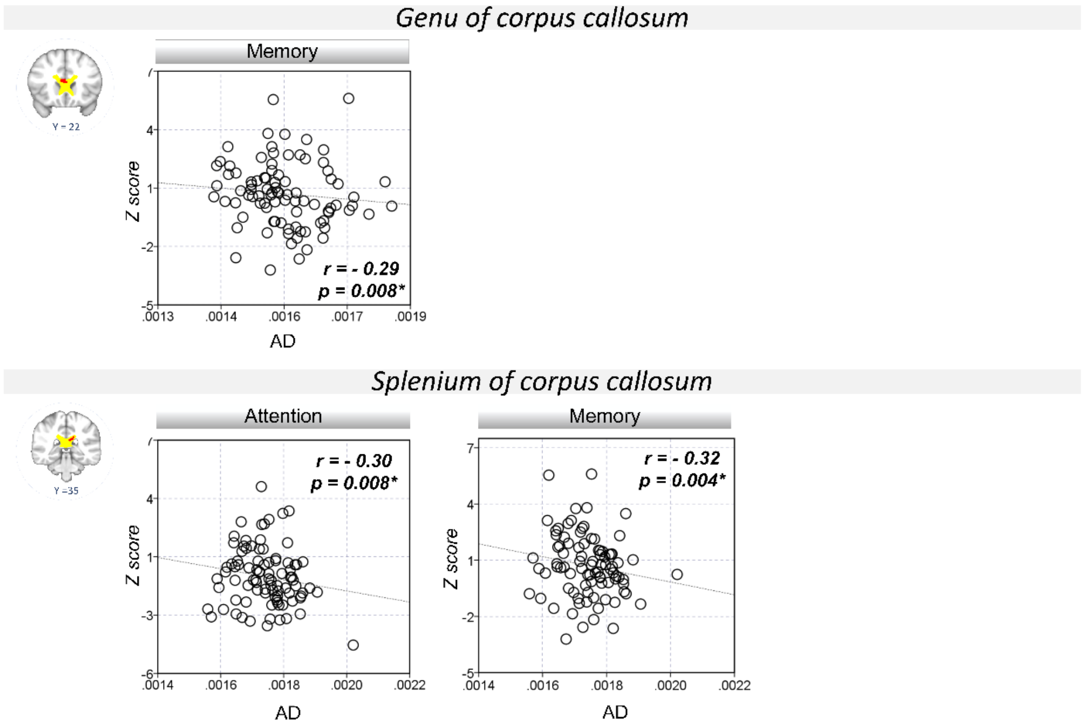

Supplementary Figure S2 | Correlations between axial diffusivity (AD) and cognitive function. The AD values of the genu of corpus callosum was correlated with memory (r = - 0.29, *P* = 0.008) while those of the splenium of corpus callosum were associated with attention (r = - 0.30, *P* = 0.008) and memory (r = - 0.32, P = 0.004) after adjusting for age, sex, education, intracranial volume, body mass index, head movements and white matter hyperintensities. **P < 0.01* as significant.

.

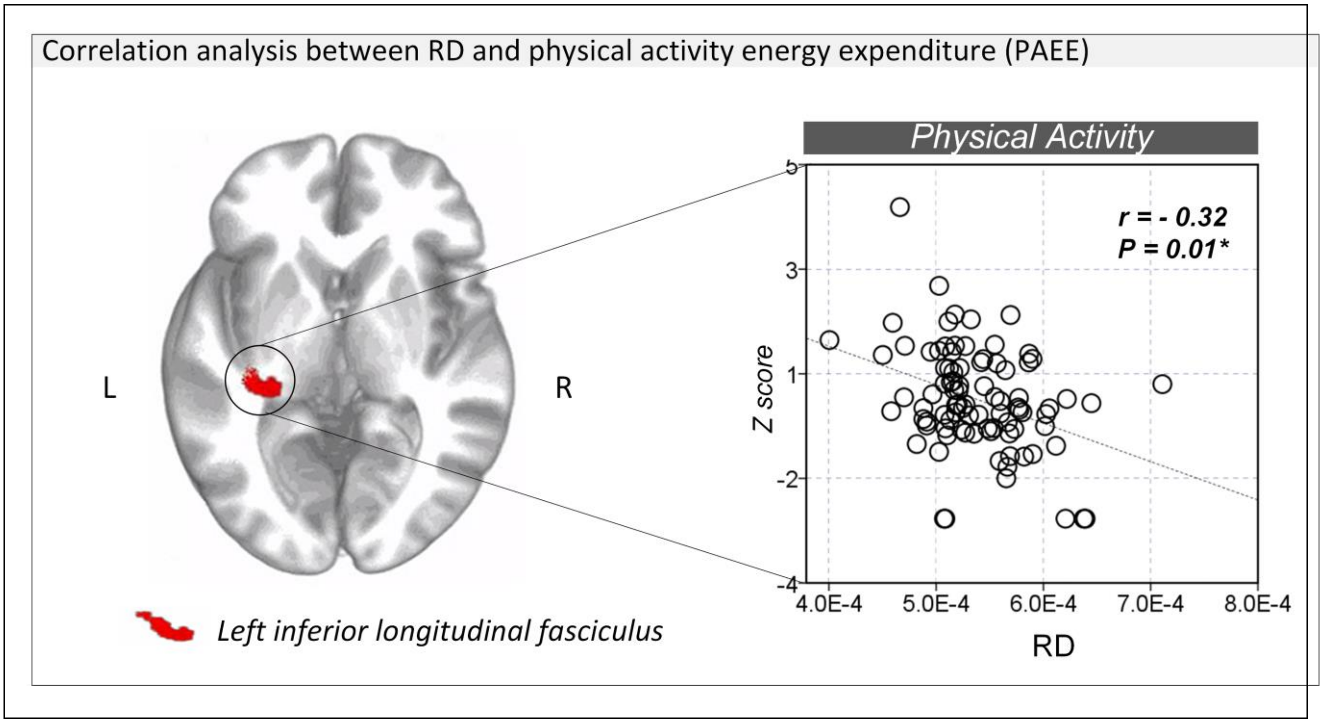

Supplementary Figure S3 | Correlation between radial diffusivity (RD) and physical activity.

A significant positive correlation between the RD values of the left inferior longitudinal fasciculus and PAEE was observed (r = -0.32, *P* = 0.01). *$P < 0.05$ as significant.
